# *In Situ* Tagged nsp15 Reveals Interactions with Coronavirus Replication/Transcription Complex-Associated Proteins

**DOI:** 10.1128/mBio.02320-16

**Published:** 2017-01-31

**Authors:** Jeremiah Athmer, Anthony R. Fehr, Matthew Grunewald, Everett Clinton Smith, Mark R. Denison, Stanley Perlman

**Affiliations:** a Department of Microbiology, University of Iowa, Iowa City, Iowa, USA; b Department of Biology, The University of the South, Sewanee, Tennessee, USA; c Department of Pediatrics, Vanderbilt University Medical Center, Nashville, Tennessee, USA; d Department of Pathology, Microbiology, and Immunology, Vanderbilt University Medical Center, Nashville, Tennessee, USA; Icahn School of Medicine at Mount Sinai

## Abstract

Coronavirus (CoV) replication and transcription are carried out in close proximity to restructured endoplasmic reticulum (ER) membranes in replication/transcription complexes (RTC). Many of the CoV nonstructural proteins (nsps) are required for RTC function; however, not all of their functions are known. nsp15 contains an endoribonuclease domain that is conserved in the CoV family. While the enzymatic activity and crystal structure of nsp15 are well defined, its role in replication remains elusive. nsp15 localizes to sites of RNA replication, but whether it acts independently or requires additional interactions for its function remains unknown. To begin to address these questions, we created an *in situ* tagged form of nsp15 using the prototypic CoV, mouse hepatitis virus (MHV). In MHV, nsp15 contains the genomic RNA packaging signal (P/S), a 95-bp RNA stem-loop structure that is not required for viral replication or nsp15 function. Utilizing this knowledge, we constructed an internal hemagglutinin (HA) tag that replaced the P/S. We found that nsp15-HA was localized to discrete perinuclear puncta and strongly colocalized with nsp8 and nsp12, both well-defined members of the RTC, but not the membrane (M) protein, involved in virus assembly. Finally, we found that nsp15 interacted with RTC-associated proteins nsp8 and nsp12 during infection, and this interaction was RNA independent. From this, we conclude that nsp15 localizes and interacts with CoV proteins in the RTC, suggesting it plays a direct or indirect role in virus replication. Furthermore, the use of *in situ* epitope tags could be used to determine novel nsp-nsp interactions in coronaviruses.

## IMPORTANCE

Despite structural and biochemical data demonstrating that the coronavirus nsp15 protein contains an endoribonuclease domain, its precise function during coronavirus infection remains unknown. In this work, we created a novel *in situ* tagged form of nsp15 to study interactions and localization during infection. This *in situ* tag was tolerated by MHV and did not affect viral replication. Utilizing this tag, we established that nsp15 localized to sites of replication but not sites of assembly throughout infection. Furthermore, we found that nsp15 interacted with the putative viral primase nsp8 and polymerase nsp12 during CoV infection. The strong association of nsp15 with replication complexes and interactions with replicative CoV enzymes suggest nsp15 is involved in CoV replication. These data and tools developed in this study help elucidate the function of nsp15 during infection and may be used to uncover other novel viral protein interactions.

## INTRODUCTION

*Coronaviridae*, members of the *Nidovirales* order, are a family of positive-sense RNA (+ssRNA) viruses that infect a wide range of host species. Generally, human coronavirus (CoV) infections cause mild disease with upper respiratory tract and gastrointestinal symptoms. In contrast, two human CoVs, severe acute respiratory syndrome (SARS)-CoV and Middle East respiratory syndrome (MERS)-CoV, recently emerged from zoonotic sources into the human population and caused severe respiratory disease with high morbidity and mortality rates (1–3). After the emergence of SARS-CoV in 2002 to 2003, efforts were made to better understand CoV replication and to develop therapies and vaccines to reduce CoV-mediated morbidity and mortality. These efforts expanded our understanding of the structure and function of several CoV proteins and of CoV replication; however, there are many aspects of the replication cycle that require further investigation (4).

Following binding and internalization of the virion, the CoV genome is deposited into the cytoplasm and translated into two large polyproteins, which account for two-thirds of the genome. These polyproteins are then cleaved by viral proteases into the nonstructural proteins nsp1 to -16. The nsps then establish a replication/transcription complex (RTC) on endoplasmic reticulum (ER) membranes, which have been restructured by viral transmembrane proteins (5, 6). To date, all studied nsps have been demonstrated to localize to replication compartments (6–12), except nsp14 and nsp16, which have not been studied. However, the precise configuration of the RTC, the binding partners of specific nsps, and the role of each nsp in replication of genomic RNA (gRNA) and transcription of subgenomic RNA (sgRNA) are not well understood.

Our current understanding of most nsp interactions comes from two-hybrid screens (13–15), cell-free *in vitro* assays (16), structural assays (17–19), or overexpression studies (11). To date, two CoV complexes containing nsp12, the RNA-dependent RNA polymerase (RdRp), have been described: (i) a complex of nsp7, nsp8, nsp12, and nsp14 demonstrated processive RNA synthesis *in vitro* (16), and (ii) a complex of nsp5, nsp8, nsp9, and nsp12 was immunoprecipitated from mouse hepatitis virus (MHV)-infected cells (9), but its function was not demonstrated. Since the majority of nsps localize to RTCs, it is likely additional interactions drive virus RNA replication and subgenomic transcription. However, due to relatively low levels of nsps produced during infection, it has been difficult to identify these interactions during a natural infection.

nsp15 contains a conserved uridine-specific endoribonuclease domain with an unknown function in CoV infection (20, 21). The endoribonuclease activity of nsp15 is conserved in CoVs and arteriviruses, but is not conserved among other nidoviruses (roniviruses and mesoniviruses) (22, 23). This lack of conservation raises the possibility that nsp15 does not function only in virus replication, but rather is also involved in immune evasion or another host-specific function. nsp15 forms a homohexamer, which is required for RNA binding and cell-free cleavage assays (24–28). CoVs and arteriviruses with endoribonuclease catalytic mutations have reduced levels of replication, as assessed by levels of infectious virus, gRNA, and subgenomic RNA (sgRNA); these effects are more pronounced in arteriviruses than in CoVs (29, 30). nsp15 was shown to colocalize with replicating RNA, but its precise localization throughout infection, interactions with other viral proteins, and physiological role are poorly understood (10).

nsp15 also contains the only known RNA packaging signal (termed “P/S” herein) in lineage A β-CoVs; this signal has been partially characterized and contains a stem-loop structure. We took advantage of the known structure of the P/S to introduce an epitope tag into nsp15 that would be useful for subsequent studies. Due to their cleavage from a larger polyprotein, N- and C-terminal nsp epitope tags are not always feasible. It was previously reported that an epitope tag inserted into the native location of nsp4 was lethal to the virus; however, the virus was viable if the epitope-tagged-nsp4 was expressed as an sgRNA (11). Notable exceptions include green fluorescent protein (GFP)-tagged nsp2 (31, 32), which is not required for replication (33), and GFP-nsp3 (32), which results in significant virus attenuation. To circumvent these problems, we inserted an influenza A virus hemagglutinin (HA) epitope tag into the P/S of MHV strain A59 (rA59_Nsp15-HA_). This HA tag was predicted to be useful for identification of protein-protein interactions and localization during infection.

The P/S is conserved among lineage A β-coronaviruses but is not present in nsp15 of other closely related β-coronaviruses and is essential for selective packaging of gRNA (34–37). Previous studies have shown that this region may be removed with no effect on viral titers, suggesting that specific amino acids in this region are not important for nsp15 function (36). rA59_Nsp15-HA_ replicated equivalently to the wild-type strain, rA59_WT_, demonstrating that this tag did not substantially affect virus replication or, by inference, significantly affect nsp15 function. We found that nsp15-HA colocalizes and interacts with CoV RTC-associated proteins nsp8 and nsp12 during infection and that this interaction was independent of RNA intermediates. Together, these data indicate that nsp15 is a component of the CoV RTC. Our data also highlight the potential utility of using internal tags to monitor the expression, localization, and interactions of CoV proteins.

## RESULTS

### Constructing recombinant MHV with HA-tagged nsp15.

**FIG 1:**
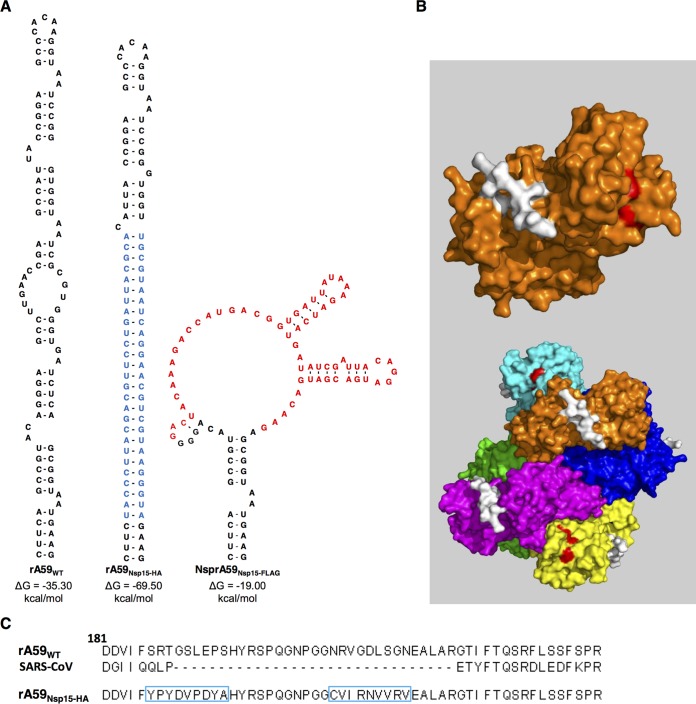
Construction of the rA59_Nsp15-HA_ *in situ* tag. (A, left) RNA secondary structure of the MHV packaging signal (38). (Middle) Mfold predicted RNA secondary structure of MHV P/S with the *in situ* HA tag and its complement (highlighted blue). (Right) Mfold predicted RNA secondary structure of Nsp15-3×FLAG with an *in situ* 3×FLAG tag (highlighted in red). (B, top) Surface rendering of an nsp15 monomer with the packaging signal highlighted in white. (Bottom) The MHV nsp15 hexamer with each monomer is depicted with a different color. Amino acids corresponding to the P/S are highlighted in white, and the catalytic triad is highlighted in red on the indicated monomer. The nsp15 crystal structure was retrieved from the PDB database (2GTH) and modified in Pymol (24). (C) MHV and SARS-CoV nsp15 protein sequence surrounding the P/S. The rA59_Nsp15_ P/S sequence with the HA sequence and its complementary sequence are boxed in blue.

To study the role of nsp15 during infection and address whether nsp15 interacts with other viral proteins, we constructed an *in situ* tagged form of nsp15 in MHV. Current antibodies used for the study of MHV nsps are limited to rabbit polyclonal sera. In many cases, these antibodies have significant background binding to host proteins, limiting their downstream applications. To circumvent this barrier, we constructed an HA-tagged form of nsp15 in MHV. In MHV, nsp15 contains the P/S, which is an ideal site for *in situ* tag insertion. The P/S, which forms a conserved stem-loop structure (Fig. 1A, left), is conserved among lineage A β-coronaviruses but not other closely related β-coronaviruses (e.g., SARS-CoV) (Fig. 1C) (38). Studies have demonstrated that the P/S can be deleted from MHV without causing significant growth defects (36). Finally, the amino acids encoded by the P/S are predicted to form a flexible loop, which is surface exposed on the monomer and hexamer of nsp15 (Fig. 1B) (24). With these characteristics in mind, we decided to design our *in situ* epitope tags around the MHV P/S.

Initially, we replaced 66 bp of the P/S with that of a 3×FLAG sequence, rA59_Nsp15-FLAG_ (Fig. 1A, right). We were able to rescue recombinant virus but were unable to detect any FLAG-specific signal by immunoblotting or immunofluorescence. Sequence analyses of the P/S revealed a near complete deletion of the 3×FLAG sequence from these viruses during initial virus replication after 5 passages (data not shown). The 3×FLAG tag was likely unsuccessful due to large differences predicted in the secondary structure and Gibbs free energy (Δ*G*) of the rA59_WT_ and the 3×FLAG-containing P/S (Fig. 1A, right). Considering these data, we set out to create an internal tag that maintained a stem-loop structure in the P/S more closely mimicking the wild-type stem-loop. To achieve this, we inserted the HA tag sequence into the ascending stem of the P/S and its complement into the descending stem of the P/S, creating rA59_Nsp15-HA_ (Fig. 1A, middle, C). A recombinant virus containing this HA tag was created using an *in vitro* ligation system as previously described (39).

### nsp15-HA is expressed and is stable during serial passaging.

**FIG 2:**
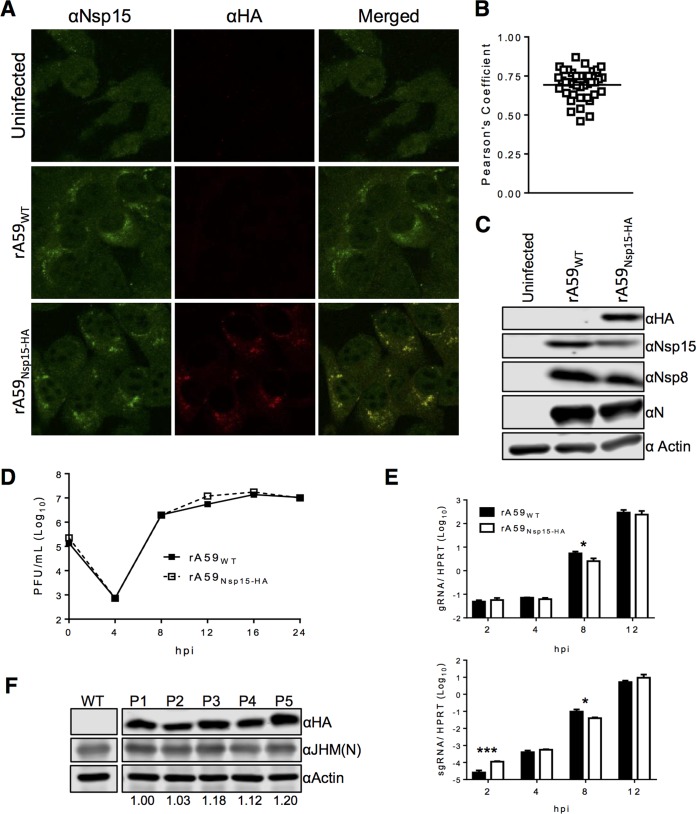
nsp15-HA protein is expressed, and the HA sequence is stable after multiple rounds of passaging *in vitro*. (A to D) 17Cl-1 cells were infected with rA59_Nsp15-HA_ or rA59_WT_ and fixed at 8 hpi. Fixed cells were costained with anti-nsp15 (green) and anti-HA (red). (B) Pearson’s correlation coefficients for at least 25 individual cells were analyzed and plotted. (C) 17Cl-1 cells were infected with rA59_Nsp15-HA_ or rA59_WT_, at an MOI of 5, and total cell lysates were harvested at 12 hpi. Cell lysates were then immunoblotted with the indicated antibodies as described in Materials and Methods. (D and E) rA59_Nsp15-HA_ has nearly identical replication kinetics to rA59_WT_ virus on 17Cl-1 cells. (D) 17Cl-1 cells were infected at an MOI of 0.1, and viral progeny were collected at the indicated time points. Input virus was collected following the adsorption step. Virus titers were determined by plaque assay on HeLa-MVR cells (E). 17Cl-1 cells were infected with rA59_WT_ or rA59_Nsp15-HA_ at an MOI of 5. Cells were collected at the indicated time points. Levels of gRNA and sgRNA were determined by RT-qPCR and normalized to hypoxanthine-guanine phosphoribosyltransferase (HPRT). All data are from a single experiment and are representative of two independent experiments. (F) rA59_Nsp15-HA_ was passaged on 17Cl-1 cells at an estimated MOI of 0.1. Virus from each passage was collected and used to infect 17Cl-1 cells for serial continued passaging and immunoblotting. Cell lysates were analyzed by immunoblotting with the indicated antibodies. The ratio of nsp15-HA to N protein was normalized to P1 and is listed below each passage. Scale bars (10 μm) are shown. Error bars indicate range (C) and standard error of the mean (SEM) (D). *, *P* < 0.05, and ***, *P* < 0.001, by Students *t* test.

Following rescue of rA59_Nsp15-HA_, we first examined the expression of nsp15-HA during MHV infection. 17Cl-1 cells were infected with rA59_Nsp15-HA_ or rA59_WT_ and analyzed by confocal microscopy and immunoblotting for HA signal. We found that anti-HA antibody could detect nsp15-HA in rA59_Nsp15-HA_-infected cells with high specificity and low background (Fig. 2A and B). Furthermore, nsp15 was localized to tight perinuclear puncta in both rA59_Nsp15-HA_- and rA59_WT_-infected cells, indicating the localization of nsp15 was not altered in rA59_Nsp15-HA_. We also detected nsp15-HA in rA59_Nsp15-HA_ cell lysates and found nsp15 levels were equivalent in rA59_Nsp15-HA_- and rA59_WT_-infected cells (Fig. 2C). These data indicated our HA tag was detectable, specific, and did not alter nsp15 expression or localization during MHV infection.

Next to examine the stability of the *in situ* HA sequence, rA59_Nsp15-HA_ was serially passaged on 17Cl-1 cells. Progeny viruses were collected at each passage and used to infect the next set of 17Cl-1 cells using a multiplicity of infection (MOI) of approximately 0.1 PFU/cell. Subsequently, each passage was analyzed for the presence of the *in situ* HA epitope tag by immunoblotting. nsp15-HA protein levels did not diminish over 5 passages (Fig. 2F), suggesting the *in situ* HA tag was stable over multiple passages, unlike the *in situ* 3×FLAG tag.

### rA59_Nsp15-HA_ has similar replication kinetics to rA59_WT_.

We next compared the kinetics of rA59_Nsp15-HA_ and rA59_WT_ replication, by infecting 17Cl-1 cells and measuring virus production under multistep growth conditions. rA59_Nsp15-HA_ replicated similarly compared to rA59_WT_ (Fig. 2D). To further support these data, 17Cl-1 cells were infected with rA59_WT_ or rA59_Nsp15-HA_, and viral gRNA and sgRNA (sgRNA7) were measured in single-step growth curves. Levels of sgRNA and gRNA in rA59_Nsp15-HA_- and rA59_WT_-infected cells differed by no more than 2-fold from 4 to 12 h postinfection (hpi) (Fig. 2E). Furthermore, amounts of both nsp8 and N proteins were equivalent in rA59_Nsp15-HA_- and rA59_WT_-infected 17Cl-1 cells (Fig. 2C). From these data, we conclude that insertion of an HA tag into the P/S within nsp15 did not substantially affect virus replication *in vitro*.

### rA59_Nsp15-HA_ has altered selective gRNA packaging.

**FIG 3:**
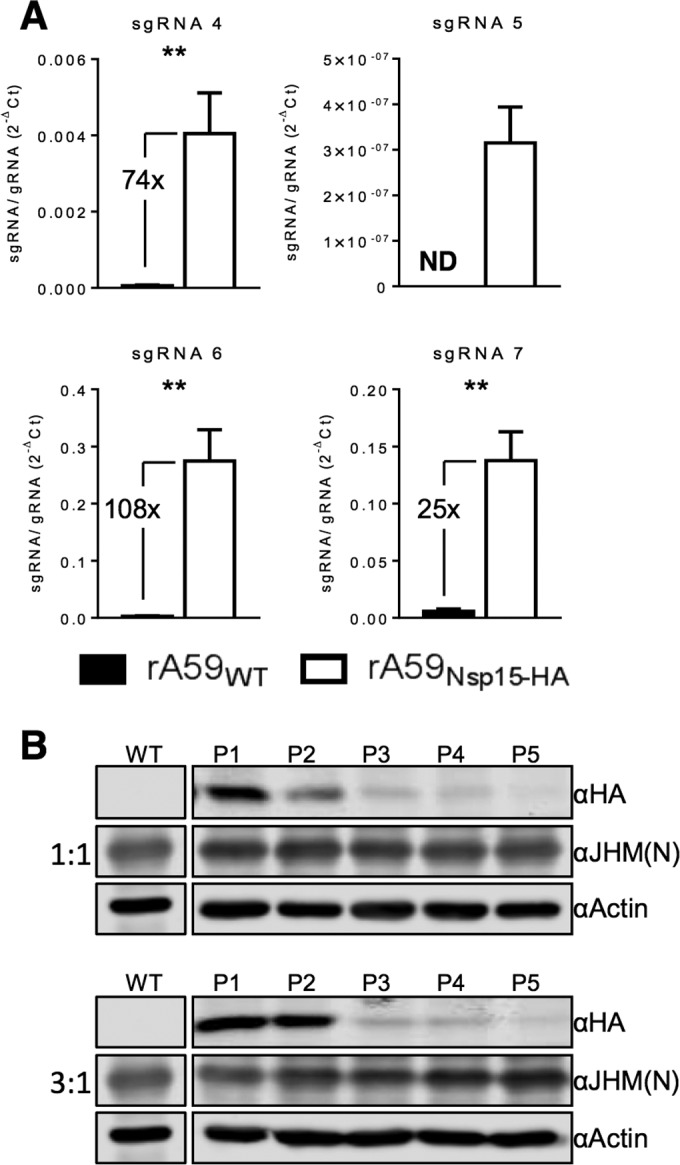
rA59_Nsp15-Ha_ virus is defective in its ability to selectively package gRNA, resulting in a loss of fitness. (A) Supernatants from rA59_Nsp15-HA_- and rA59_WT_-infected 17Cl-1 cells were collected, and cell debris was removed. Virions were pelleted by ultracentrifugation through a 30% sucrose cushion, and viral RNA was isolated. The ratio of sgRNAs to gRNA in viral RNA was measured by RT-qPCR. (B) 17Cl-1 cells were infected with the indicated ratios of rA59_Nsp15-HA_ to rA59_WT_, with a total MOI of 0.1. Progeny viruses were then passaged at an estimated MOI of 0.1 and collected. Progeny virus from each passage was used to infect 17Cl-1 cells at an MOI of 0.1. Cell lysates were collected at 16 hpi and immunoblotted with the indicated antibodies. Figures 2D and 3B were analyzed in parallel and are imaged from the same immunoblot. **, *P* < 0.01 by Mann-Whitney *U* test.

During our investigation of viral growth kinetics, we noted that rA59_Nsp15-HA_ had significantly increased levels of sgRNA at 2 hpi. We hypothesized that this increase in sgRNA levels was too early to be due to sgRNA transcription and attributed it to increased packaging of sgRNA into virions by rA59_Nsp15-HA_. This would be in agreement with previous reports, which demonstrated that P/S mutants altered selective gRNA packaging, so that sgRNA was now incorporated into virions, but did not affect viral replication (36). To determine the efficiency of selective packaging in rA59_Nsp15-HA_, we measured the ratio of sgRNA (sgRNAs 4, 5, 6, and 7) to gRNA in virus isolated from infected cell supernatants by ultracentrifugation. Our results showed that viral RNA obtained from rA59_Nsp15-HA_-infected cell supernatants had at least a 25-fold increase in the sgRNA/gRNA ratio (Fig. 3A). Previous work demonstrated that P/S mutants and wild-type virus have identical growth kinetics, but P/S mutants are readily outcompeted by rA59_WT_ when cells are dually infected (36). In order to test whether the rA59_Nsp15-HA_ virus would be outcompeted by rA59_WT_ virus, cells were infected with 1:1 or 3:1 mixtures of rA59_Nsp15-HA_ and rA59_WT_ at a total MOI of 0.1. This low MOI was used to ensure that only a small number of cells would be infected by both viruses. At 16 hpi, progeny viruses were collected and subsequently used for both immunoblotting and further passaging. The rA59_Nsp15-HA_ was rapidly outcompeted by rA59_WT_, as nsp15-HA signal was greatly diminished by passages 2 and 3 for 1:1 and 3:1 infection ratios, respectively (Fig. 3B). Taken together, these data indicated that while rA59_Nsp15-HA_ replicates normally, the HA insertion significantly hampers its capacity to selectively package gRNA over sgRNA, diminishing its competitive advantage. This suggests that while the *in situ* tag maintained a stem-loop within this region of nsp15, it slightly attenuated the virus by likely altering the primary sequence of the P/S or the internal secondary structure of the RNA in this region or, possibly, a function of nsp15.

### nsp15 colocalizes with RTC members nsp8 and nsp12.

**FIG 4:**
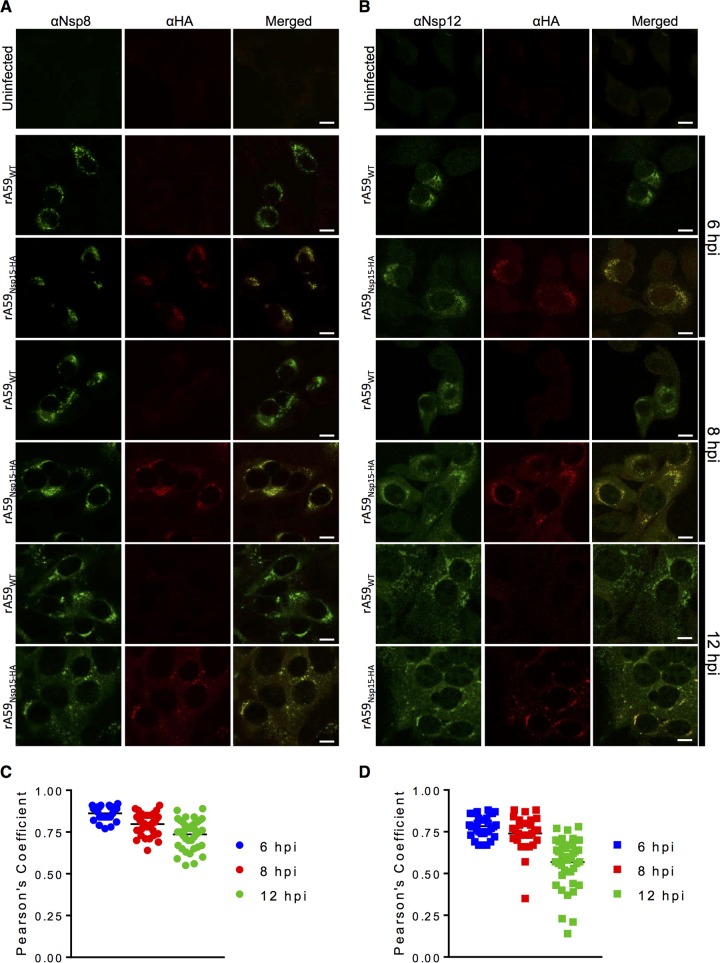
nsp15 strongly colocalizes with nsp8 and nsp12 during infection. (A and B) Uninfected 17Cl-1 cells were costained with anti-nsp8 (green) and anti-HA (red) (A) or anti-nsp12 (green) and anti-HA (red) (B). (C and D) 17Cl-1 cells were infected with rA59_Nsp15-HA_ or rA59_WT_ at an MOI of 1. Cells were fixed and stained at 6 (top), 8 (middle), or 12 (bottom) hpi and costained with either anti-HA and anti-nsp8 (C) or anti-HA and anti-nsp12 (D). Each image is representative of at least 50 cells from two independent experiments. Pearson’s correlation coefficients for at least 25 individual cells were analyzed for each time point for nsp8 (E) and nsp12 (F) and plotted. Scale bars (10 μm) are shown.

Previously, it was shown that nsp15 localizes with replicating viral RNA (10), potentially to replication compartments and with RTC members. To confirm that nsp15 was in fact localized to RTCs and to expand upon these results, we investigated the colocalization of nsp15 with nsp8 and nsp12 throughout infection. nsp8 and nsp12, the proposed viral primase and RdRp, respectively, are both established members of the replication complex and often used as markers for RTC localization (8, 9). Following infection with either rA59_Nsp15-HA_ or rA59_WT_, 17Cl-1 cells were fixed at indicated time points throughout infection and then costained with anti-HA and anti-nsp8 or anti-nsp12 antibody. nsp15 strongly colocalized with both nsp8 and nsp12 in rA59_Nsp15-HA_-infected cells throughout infection (Fig. 4). The average Pearson’s correlation coefficients (PCCs) ranged from 0.84 to 0.71 for nsp8 and 0.78 to 0.53 for nsp12. There was a small decrease in PCCs for both nsp8 and nsp12 at later time points; however, the PCCs remained above 0.5 throughout infection, which is considered strong colocalization. This colocalization was present in both syncytia and individual infected cells (Fig. 4A and B). These data demonstrated that nsp15 strongly colocalized with two proteins associated with the CoV RTC by confocal microscopy.

### nsp15 does not localize to sites of assembly.

**FIG 5:**
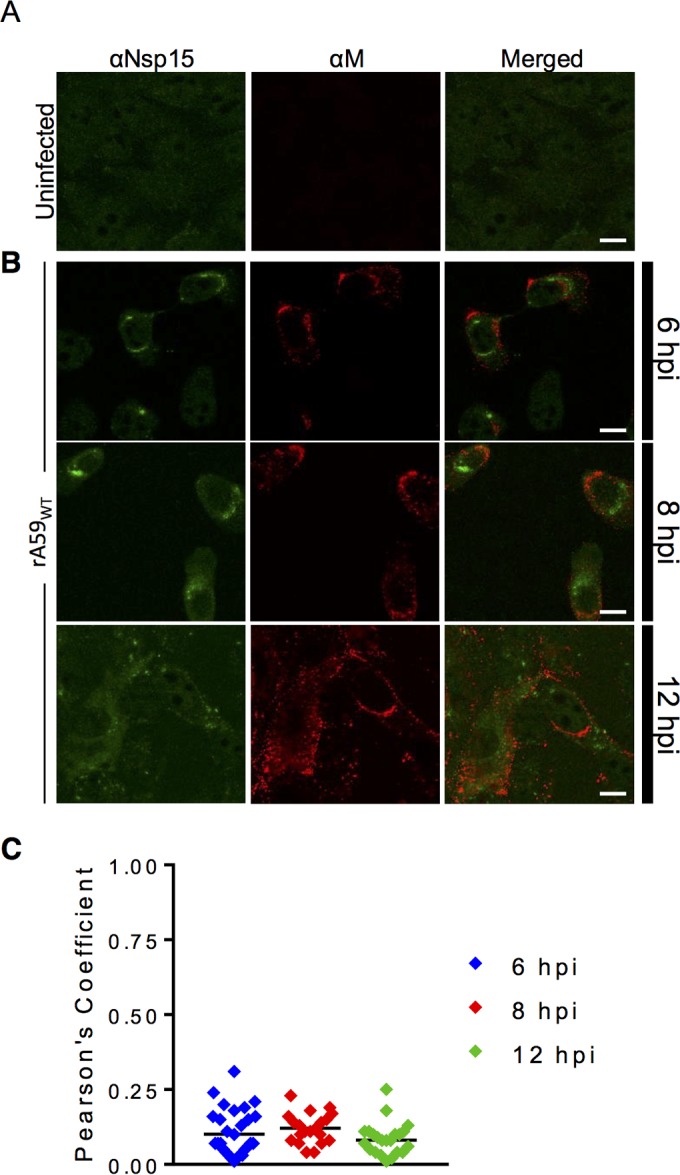
nsp15 does not localize to sites of assembly during infection. (A) Uninfected 17Cl-1 cells were costained with anti-nsp15 (green) and anti-M (red). (B) 17Cl-1 cells were infected with rA59_WT_ at an MOI of 1. Cells were fixed and stained at 6 (top), 8 (middle), or 12 (bottom) hpi and costained with anti-nsp15 (green) and anti-M protein (red). (C) Pearson’s correlation coefficients for at least 25 individual cells were analyzed for each time point and plotted.

These results demonstrated strong colocalization of nsp15 with RTCs but did not address whether nsp15 also localized to sites of virus assembly. To this end, we costained rA59_WT_-infected cells with anti-nsp15 and anti-M protein antibodies. M protein is localized to the endoplasmic reticulum-Golgi intermediate compartment (ERGIC) and is a marker for sites of assembly (40). To more formally confirm a lack of colocalization, we used rabbit anti-nsp15 to enable the use of a mouse monoclonal antibody (MAb) to M protein. M protein displayed tight punctate staining, most likely in the ERGIC compartment, at 6 and 8 hpi (Fig. 5B, top and middle). At 12 hpi, most cells were syncytial, and M puncta were scattered throughout the cytoplasm and potentially the cell membrane, most likely due to Golgi fragmentation (Fig. 5B, bottom) (41). At all stages of infection, nsp15 did not colocalize with M protein (Fig. 5B), resulting in low PCCs of <0.3 throughout infection (Fig. 5C). Similar results were obtained when cells were infected with rA59_Nsp15-HA_ in lieu of rA59_WT_ (data not shown). These results indicated that nsp15 was not localized to sites of assembly during infection.

### nsp15 interacts with CoV RTC-associated proteins.

**FIG 6:**
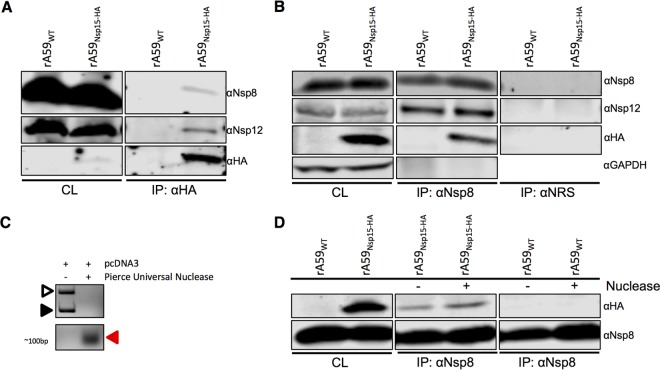
nsp15 interacts with the RTC proteins nsp8 and nsp12 in an RNA-independent manner. (A, B, and D). 17Cl-1 cells were infected with rA59_Nsp15-HA_ or rA59_WT_ at an MOI of 0.1 and collected at 20 hpi. Cell lysates were subjected to immunoprecipitation with anti-HA (A), anti-nsp8 (B and D), or normal rabbit serum (NRS) (B). Cell lysates (CL) and eluted proteins were immunoblotted with the indicated antibodies. (C) Lysis buffer was spiked with 3 μg of pcDNA3 plasmid with or without nuclease (250 U) and incubated for 8 h. The DNA was analyzed by agarose gel electrophoresis and visualized with ethidium bromide. The solid arrowhead represents linearized plasmid DNA, and the open arrowhead represents the supercoiled DNA. The red arrowhead indicates degraded products of ~100 bp. (D) 17Cl-1 cells were infected as described above, and cell lysates were subjected to immunoprecipitation as described in panel B in the presence or absence of Pierce Universal Nuclease.

Due to the strong colocalization of nsp15 with nsp8 and nsp12, we next investigated whether nsp15 physically interacted with these proteins. 17Cl-1 cells were infected with rA59_Nsp15-HA_ or rA59_WT_, and lysates were incubated with HA or nsp8 antibodies to identify interacting partners. Using the anti-HA antibody, no proteins were immunoprecipitated in rA59_WT_-infected cells, while nsp8, nsp12, and nsp15-HA coprecipitated in rA59_Nsp15-HA_-infected cells (Fig. 6A). To confirm these interactions, we next examined whether anti-nsp8 antibody would coprecipitate nsp15-HA. We found that nsp12 coprecipitated with nsp8 in both rA59_Nsp15-HA_- and rA59_WT_-infected cells, in agreement with previous results (Fig. 6B) (9). Furthermore, we found that nsp15-HA protein was immunoprecipitated with nsp8 antibody only in rA59_Nsp15-HA_-infected cells (Fig. 6B). In these experiments, no precipitation of the irrelevant control protein GAPDH (glyceraldehyde-3-phosphate dehydrogenase) was observed, and no viral proteins were immunoprecipitated with normal rabbit serum (Fig. 6B). Taken together, these data demonstrated that nsp15 interacted with RTC proteins during MHV infection.

Since nsp8, nsp12, and nsp15 are all RNA binding or processing enzymes, we next investigated the dependence of these interactions on RNA binding. To accomplish this, we added a general RNA/DNA nuclease (Pierce Universal Nuclease), which we showed was active in our immunoprecipitation (IP) buffer (Fig. 6C), to cleave all unprotected RNA. We found no difference in the interactions between nsp8 and nsp15-HA when nuclease was present (Fig. 6D), demonstrating that these interactions were not dependent on RNA intermediates.

## DISCUSSION

While nsp15 is completely conserved among CoVs and is known to have endoribonuclease activity *in vitro*, the role of this protein during infection is still largely unknown. nsp15 is likely to have a role in enhancing virus replication since mutation of catalytic amino acids resulted in reduced accumulation of viral RNA (29). Further, it was previously shown that nsp15 localized with replicating RNA (10), but its localization and interactions with other viral proteins were previously unknown. In this work, utilizing our *in situ* HA tag, we found strong colocalization of nsp15 with nsp8 and nsp12 throughout infection (Fig. 4). Further, we found only low levels of staining outside the RTC and no colocalization with M protein (Fig. 5). These data indicate that nsp15 localized to replication compartments with little or no detectable localization to other cellular or viral compartments. We found that nsp15 interacted with two markers of the RTC, nsp8 and nsp12, both with and without nuclease treatment, indicating that these interactions occur without the involvement of an RNA intermediate (Fig. 6). Our methods cannot distinguish direct from indirect interactions, so it remains possible that a viral or host protein mediates these binding events. While these interactions were detected in MHV-infected cells, a previous yeast two-hybrid (Y2H) screen along with a glutathione *S*-transferase (GST) pulldown identified potential binding partners of SARS-CoV nsp15, including nsp8 and nsp12 (15), suggesting these interactions are conserved among CoVs. These data collectively suggest that nsp15 is required for optimal viral production. However, further work will be required to determine if this occurs by directly or indirectly enhancing CoV RNA synthesis, processing RNA intermediates, or countering host cell defenses.

Incubation with anti-HA or anti-nsp8 antibody only coprecipitated a small fraction of the respective binding partner (Fig. 6). We hypothesize that this could be due to one or a combination of the following possibilities: (i) only a small percentage of viral nsps are engaged in RNA replication at any point in time as reported for hepatitis C virus (42), (ii) nsp15 has multiple functions, only one of which requires interaction with other nsps, or (iii) nsp15 only transiently interacts with the RTC before completing its function and subsequently dissociates from the complex. Previous data have demonstrated a link between nsp15’s oligomeric state and RNA binding and cleavage (24–26). Oligomer formation was required for binding and cleavage *in vitro*, and cycling of nsp15 between a hexamer and a monomer was hypothesized to be required for RNA cleavage (25). Thus, nsp15 could be available for binding to RTC components intermittently.

In the present study, both the sequence and structure of the P/S stem were altered by our *in situ* tag. While viral replication was unaffected, viral fitness was modestly decreased compared to wild-type virus (Fig. 3B). This decrease in viral fitness was most likely due to a significant decrease in selective gRNA packaging in rA59_Nsp15-HA_, as a similar phenotype was described with a P/S deletion virus (36). In rA59_Nsp15-HA_, we found a significant >25-fold (Fig. 3A) increase in sgRNA packaging. This decrease in selective packaging may reflect requirements for the primary sequence of the P/S stem, proper secondary structure, or a combination of both, but at this point, we cannot distinguish between these possibilities. It has been hypothesized that internal conserved secondary structures, e.g., 2-bp bulges, play a functional role in selective packaging (38). From our data, we hypothesize that the pentaloop and the remaining 2-bp bulge are not sufficient for efficient selective packaging. However, it remains possible that they are necessary for some degree of selective packaging, perhaps mediating interactions with M protein (34). This may explain why the HA tag sequence was stable, whereas the FLAG tag sequence was quickly lost following passaging of the virus (Fig. 3B; data not shown). Further work is required to determine what factors within the P/S are important for selective packaging of genomic RNA.

In conclusion, we demonstrated that the use of internal tags is tolerated by the MHV polyprotein and suggest that this approach may be generally useful for defining CoV RTC components and for identifying other interactions within the RTC. To create the *in situ* tag, we first identified a site with favorable characteristics for the insertion of a tag. These characteristics include (i) lack of conservation in other β-CoVs, suggesting that the site would tolerate mutation, (ii) being nonessential for replication, and (iii) surface exposure. The MHV P/S located within nsp15 met all of these criteria. We found that our rA59_Nsp15-HA_ *in situ* tag had little to no effect on viral RNA transcription/replication, viral protein expression, or virus production (Fig. 2). Using criteria similar to those presented here, we believe other CoV proteins could be internally tagged and studied during infection from native expression. The creation of internal tags and use of their corresponding monoclonal antibody (MAb) open new applications for the study of CoV replication, most notably the identification of novel *in vivo* RTC interactions.

## MATERIALS AND METHODS

### Cell culture.

17Cl-1 cells, HeLa cells expressing MHV receptor carcinoembryonic antigen-related cell adhesion molecule 1 (CEACAM1) (43), (HeLa-MVR), and baby hamster kidney cells expressing CEACAM1 (BHK-R) (44) were grown as previously described (39, 45).

### Generation of recombinant strain rA59_Nsp15-HA_.

An epitope derived from the influenza A virus hemagglutinin (HA), YPYDVPDYA, was introduced into the F plasmid of the A59 *in vitro* ligation system (39). The F plasmid was linearized, and homologous arms, with the HA substitution, were added by PCR using the following primers: 5′ GAGCCCACAAGGTAATCCGGGTGGTTGCGTAATCAGGAACGTCGTAAGGGTAGAAGCTCTAGCGCGTGGCAC 3′ and 5′ GATTACCTTGTGGGCTCCGGTAATGTGCGTAATCAGGAACGTCGTAAGGGTAGAAGATAACATCGTCACCGT 3′. The plasmid was recombined using In-Fusion (Clontech) according to the manufacturer’s protocol and screened for the HA substitution. MHV-A59 fragments were digested and ligated as previously reported (46). Transcripts were generated using mMessage Machine T7. RNAs were then electroporated (Bio-Rad GenePulser Xcell) into 6 × 10^7^ BHK-R cells seeded into a T75 flask. Flasks were monitored for cytopathic effect (CPE [24 to 48 h]), and viruses were collected by freeze-thawing at peak CPE. After thawing, cell debris was cleared by centrifugation at 1,500 × *g*, and supernatants were then aliquoted and termed passage 0 (P0). To create working stocks, rA59_Nsp15-HA_ and rA59_WT_ P0 viruses were passaged on 17Cl-1 cells and collected at maximum CPE.

### Virus infection.

Recombinant versions of the A59 strain of MHV (termed rA59 herein) were used in all experiments. The cDNA clone used to develop recombinant rA59_Nsp15-HA_ and rA59_WT_ has been described previously (GenBank accession no. AY910861) (39). Unless otherwise stated, 17Cl-1 cells were infected with the indicated viruses at an MOI of 0.1 for 30 min at 37°C. Viruses from supernatants and cells were combined prior to determination of viral titers.

### Viral titers.

Virus titers were determined on HeLa-MVR cells as previously described with some modifications (47). HeLa-MVR cells under agarose overlays were fixed with 3.7% formaldehyde at 16 to 24 hpi and stained with 0.1% crystal violet.

### Passaging and competition assay.

The rA59_Nsp15-HA_ P1 viruses were passaged on 17Cl-1 cells at an initial MOI of 0.1. Each passage was collected at maximum CPE. For each subsequent passage, an estimated MOI of 0.1 was used by infecting 17Cl-1 cells with 5 μl of infected supernatants. Following the fifth passage, stocks of each virus were used to infect 17Cl-1 cells and collected for immunoblotting as described below.

### RNA analysis.

Subconfluent monolayers of 17Cl-1 cells were infected at an MOI of 5 and collected by Trizol (Thermo Fisher Scientific) at the indicated times. RNA was isolated according to the manufacturer’s instructions and transcribed into cDNA using Moloney murine leukemia virus reverse transcriptase (MMLV RT) (Thermo Fischer Scientific). Viral gRNA, sgRNA, and cellular hypoxanthine phosphoribosyltransferase (HPRT) RNA levels were measured by quantitative PCR (qPCR) with the indicated primers (see Table S1 in the supplemental material) using an Applied Biosystems 7300 real-time PCR system (Applied Biosystems). The levels of gRNA and sgRNA were normalized to HPRT by the following threshold cycle (*C*_*T*_) equation: Δ*C*_*T*_ = *C*_*T*_ of gene of interest − C_*T*_ of HPRT. All results are shown as a ratio to HPRT calculated as 2^−Δ*CT*^.

10.1128/mBio.02320-16.1 TABLE S1  List of primers used in this study. Download TABLE S1, DOCX file, 0.1 MB. Copyright © 2017 Athmer et al. 2017 Athmer et al. This content is distributed under the terms of the Creative Commons Attribution 4.0 International license.

### Selective viral RNA packaging.

Cell supernatants were collected at 16 hpi from 1.5 × 10^7^ 17Cl-1 cells infected with rA59_WT_ or rA59_Nsp15-HA_ at an MOI of 0.1. Supernatants were centrifuged at 1,000 rpm to remove cellular debris and then passed through a 45-µm-pore filter. Viruses were pelleted through a 30% sucrose cushion for 4 h at 27,000 rpm using a Beckman ultracentrifuge and an SW32 Ti rotor (Beckman, Indianapolis, IN). Viral pellets were resuspended in Dulbecco’s modified Eagle’s medium (DMEM), and viral RNA was isolated using Trizol. The ratio of sgRNA to gRNA was measured using reverse transcription-quantitative PCR (RT-qPCR).

### Confocal microscopy.

Subconfluent monolayers of 17Cl-1 cells on glass coverslips were infected at an MOI of 1 with either rA59_WT_ or rA59_Nsp15-HA_ virus. At the indicated times, monolayers were fixed with 4% paraformaldehyde. Cells were then permeabilized with 0.1% Triton X-100 in phosphate-buffered saline (PBS) before blocking with 1% goat serum. Following blocking, cells were stained with indicated primary antibodies for 3 h at room temperature using the following concentrations: anti-HA, 1:500 (clone 16B12 [BioLegend]); anti-nsp8, 1:1,000 (8); anti-nsp12, 1:500 (9); anti-M (5B11.5), 1:10,000 (48); and anti-nsp15 (D23 [a generous gift from Susan Baker, Loyola University, Chicago, IL]), 1:500 (10, 49). Following washing with blocking buffer, fluorophore-conjugated secondary antibody was applied. Goat anti-rabbit 488 and goat anti-mouse 568 (Thermo Fisher Scientific) were used 1:500 in blocking buffer for 1 h at room temperature. Cells were washed before mounting with Vectashield antifade reagent (Vector Laboratories). In most cases, 4 to 5 images, with an average of ~10 cells per image for each condition and for each experiment, were taken using a Leica STED Sp8 scanning confocal microscope. Images were analyzed using LAS X software. Pearson’s correlation coefficients (PCCs) were obtained by analyzing individual cells from at least four images from two independent experiments using the CoLoc2 plugin for FIJI (50). Background was subtracted for data analysis as required.

### Immunoblotting.

Infected or uninfected subconfluent monolayers of 17Cl-1 cells were lysed with 2× sample buffer containing SDS, protease and phosphatase inhibitors (Roche, Basal, Switzerland), β-mercaptoethanol, and Pierce Universal Nuclease (Thermo Fisher Scientific). Boiled cell lysates were separated by SDS-PAGE on polyacrylamide gels. Gels were transferred overnight at 30 V to polyvinylidene difluoride (PVDF) membranes (Millipore), and the membranes were blocked with 5% milk in PBS or Odyssey blocking buffer (Li-Cor). Blots were incubated with listed primary antibodies, for 3 h at room temperature: anti-nsp8, 1:1,000; anti-nsp12, 1:500; anti-HA, 1:500; anti-N protein (5B188.2), 1:10,000 (a generous gift from Michael Buchmeier, University of California Irvine); anti-JHM, 1:10,000 (51); and anti-actin, 1:10,000 (clone AC-15 [Abcam, Inc.]). Immunoblots were washed with 0.1% Tween in PBS. Blots were incubated with infrared-conjugated secondary antibodies (Li-Cor) for 1 h and then washed with PBS before imaging. Immunoblots were imaged using a Li-Cor Odyssey Imager and analyzed using Image Studio software (Li-Cor).

### Immunoprecipitation.

17Cl-1 cells were plated on 10-cm dishes 2 days prior to infection. For each condition, 4.5 × 10^7^ 17Cl-1 cells were infected with either rA59_WT_ or rA59_Nsp15-HA_ at an MOI of 0.1. When plates reached maximum CPE (16 to 20 hpi), cells were collected and pelleted by low-speed centrifugation prior to lysis. Cell pellets were lysed with IP buffer (0.5% NP-40, 150 mM NaCl, 5% glycerol, and 50 mM Tris [pH 8.0], with or without Pierce Universal Nuclease [Thermo Fischer Scientific]), as indicated for 1 h at 4°C. Next, nuclei were pelleted by centrifugation (16,000 × *g* for 15 min at 4°C) and applied to protein G Dynabeads (Thermo Fischer Scientific) conjugated to the indicated antibodies according to the manufacturer’s protocol. Cell lysate and antibody-bound beads were mixed overnight at 4°C. Beads were washed with PBS-Tween before elution with 2× SDS sample buffer at 95°C for 5 min. Eluates and lysates were then subjected to immunoblotting as described above.

### Statistics.

Student’s unpaired *t* test or Mann-Whitney *U* test was used to analyze differences in mean values between groups. All results are expressed as means ± range or standard error of the mean. *P* values of ≤0.05 were considered statistically significant.

## References

[1] Fehr AR, Channappanavar R, Perlman S 2016 Middle East respiratory syndrome: emergence of a pathogenic human coronavirus. Annu Rev Med. doi:10.1146/annurev-med-051215-031152. PMC5353356 27576010

[2] Hilgenfeld R, Peiris M 2013 From SARS to MERS: 10 years of research on highly pathogenic human coronaviruses. Antiviral Res 100:286–295. doi:10.1016/j.antiviral.2013.08.015. 24012996 PMC7113673

[3] Perlman S, Netland J 2009 Coronaviruses post-SARS: update on replication and pathogenesis. Nat Rev Microbiol 7:439–450. doi:10.1038/nrmicro2147. 19430490 PMC2830095

[4] Neuman BW, Chamberlain P, Bowden F, Joseph J 2014 Atlas of coronavirus replicase structure. Virus Res 194:49–66. doi:10.1016/j.virusres.2013.12.004. 24355834 PMC7114488

[5] Angelini MM, Akhlaghpour M, Neuman BW, Buchmeier MJ 2013 Severe acute respiratory syndrome coronavirus nonstructural proteins 3, 4, and 6 induce double-membrane vesicles. mBio 4:e00524-13. doi:10.1128/mBio.00524-13. PMC3747587 23943763

[6] Knoops K, Kikkert M, Worm SH, Zevenhoven-Dobbe JC, van der Meer Y, Koster AJ, Mommaas AM, Snijder EJ 2008 SARS-coronavirus replication is supported by a reticulovesicular network of modified endoplasmic reticulum. PLoS Biol 6:e226. doi:10.1371/journal.pbio.0060226. 18798692 PMC2535663

[7] van der Meer Y, Snijder EJ, Dobbe JC, Schleich S, Denison MR, Spaan WJ, Locker JK 1999 Localization of mouse hepatitis virus nonstructural proteins and RNA synthesis indicates a role for late endosomes in viral replication. J Virol 73:7641–7657. 10438855 10.1128/jvi.73.9.7641-7657.1999 PMC104292

[8] Bost AG, Carnahan RH, Lu XT, Denison MR 2000 Four proteins processed from the replicase gene polyprotein of mouse hepatitis virus colocalize in the cell periphery and adjacent to sites of virion assembly. J Virol 74:3379–3387. doi:10.1128/JVI.74.7.3379-3387.2000. 10708455 PMC111839

[9] Brockway SM, Clay CT, Lu XT, Denison MR 2003 Characterization of the expression, intracellular localization, and replication complex association of the putative mouse hepatitis virus RNA-dependent RNA polymerase. J Virol 77:10515–10527. doi:10.1128/JVI.77.19.10515-10527.2003. 12970436 PMC228489

[10] Shi ST, Schiller JJ, Kanjanahaluethai A, Baker SC, Oh JW, Lai MM 1999 Colocalization and membrane association of murine hepatitis virus gene 1 products and de novo-synthesized viral RNA in infected cells. J Virol 73:5957–5969. 10364348 10.1128/jvi.73.7.5957-5969.1999 PMC112657

[11] Hagemeijer MC, Ulasli M, Vonk AM, Reggiori F, Rottier PJ, de Haan CA 2011 Mobility and interactions of coronavirus nonstructural protein 4. J Virol 85:4572–4577. doi:10.1128/JVI.00042-11. 21345958 PMC3126240

[12] Brockway SM, Lu XT, Peters TR, Dermody TS, Denison MR 2004 Intracellular localization and protein interactions of the gene 1 protein p28 during mouse hepatitis virus replication. J Virol 78:11551–11562. doi:10.1128/JVI.78.21.11551-11562.2004. 15479796 PMC523235

[13] Pan J, Peng X, Gao Y, Li Z, Lu X, Chen Y, Ishaq M, Liu D, Dediego ML, Enjuanes L, Guo D 2008 Genome-wide analysis of protein-protein interactions and involvement of viral proteins in SARS-CoV replication. PLoS One 3:e3299. doi:10.1371/journal.pone.0003299. 18827877 PMC2553179

[14] von Brunn A, Teepe C, Simpson JC, Pepperkok R, Friedel CC, Zimmer R, Roberts R, Baric R, Haas J 2007 Analysis of intraviral protein-protein interactions of the SARS coronavirus ORFeome. PLoS One 2:e459. doi:10.1371/journal.pone.0000459. 17520018 PMC1868897

[15] Imbert I, Snijder EJ, Dimitrova M, Guillemot JC, Lécine P, Canard B 2008 The SARS-Coronavirus PLnc domain of nsp3 as a replication/transcription scaffolding protein. Virus Res 133:136–148. doi:10.1016/j.virusres.2007.11.017. 18255185 PMC7114086

[16] Subissi L, Posthuma CC, Collet A, Zevenhoven-Dobbe JC, Gorbalenya AE, Decroly E, Snijder EJ, Canard B, Imbert I 2014 One severe acute respiratory syndrome coronavirus protein complex integrates processive RNA polymerase and exonuclease activities. Proc Natl Acad Sci U S A 111:E3900–E3909. doi:10.1073/pnas.1323705111. PMC4169972 25197083

[17] Zhai Y, Sun F, Li X, Pang H, Xu X, Bartlam M, Rao Z 2005 Insights into SARS-CoV transcription and replication from the structure of the nsp7-nsp8 hexadecamer. Nat Struct Mol Biol 12:980–986. doi:10.1038/nsmb999. 16228002 PMC7096913

[18] Decroly E, Debarnot C, Ferron F, Bouvet M, Coutard B, Imbert I, Gluais L, Papageorgiou N, Sharff A, Bricogne G, Ortiz-Lombardia M, Lescar J, Canard B 2011 Crystal structure and functional analysis of the SARS-coronavirus RNA cap 2′-O-methyltransferase nsp10/nsp16 complex. PLoS Pathog 7:e1002059. doi:10.1371/journal.ppat.1002059. 21637813 PMC3102710

[19] Ma Y, Wu L, Shaw N, Gao Y, Wang J, Sun Y, Lou Z, Yan L, Zhang R, Rao Z 2015 Structural basis and functional analysis of the SARS coronavirus nsp14-nsp10 complex. Proc Natl Acad Sci U S A 112:9436–9441. doi:10.1073/pnas.1508686112. 26159422 PMC4522806

[20] Bhardwaj K, Guarino L, Kao CC 2004 The severe acute respiratory syndrome coronavirus Nsp15 protein is an endoribonuclease that prefers manganese as a cofactor. J Virol 78:12218–12224. doi:10.1128/JVI.78.22.12218-12224.2004. 15507608 PMC525082

[21] Ivanov KA, Hertzig T, Rozanov M, Bayer S, Thiel V, Gorbalenya AE, Ziebuhr J 2004 Major genetic marker of nidoviruses encodes a replicative endoribonuclease. Proc Natl Acad Sci U S A 101:12694–12699. doi:10.1073/pnas.0403127101. 15304651 PMC514660

[22] Nga PT, Parquet Mdel C, Lauber C, Parida M, Nabeshima T, Yu F, Thuy NT, Inoue S, Ito T, Okamoto K, Ichinose A, Snijder EJ, Morita K, Gorbalenya AE 2011 Discovery of the first insect nidovirus, a missing evolutionary link in the emergence of the largest RNA virus genomes. PLoS Pathog 7:e1002215. doi:10.1371/journal.ppat.1002215. 21931546 PMC3169540

[23] Nedialkova DD, Ulferts R, van den Born E, Lauber C, Gorbalenya AE, Ziebuhr J, Snijder EJ 2009 Biochemical characterization of arterivirus nonstructural protein 11 reveals the nidovirus-wide conservation of a replicative endoribonuclease. J Virol 83:5671–5682. doi:10.1128/JVI.00261-09. 19297500 PMC2681944

[24] Xu X, Zhai Y, Sun F, Lou Z, Su D, Xu Y, Zhang R, Joachimiak A, Zhang XC, Bartlam M, Rao Z 2006 New antiviral target revealed by the hexameric structure of mouse hepatitis virus nonstructural protein nsp15. J Virol 80:7909–7917. doi:10.1128/JVI.00525-06. 16873248 PMC1563835

[25] Guarino LA, Bhardwaj K, Dong W, Sun J, Holzenburg A, Kao C 2005 Mutational analysis of the SARS virus Nsp15 endoribonuclease: identification of residues affecting hexamer formation. J Mol Biol 353:1106–1117. doi:10.1016/j.jmb.2005.09.007. 16216269 PMC7094243

[26] Ricagno S, Egloff MP, Ulferts R, Coutard B, Nurizzo D, Campanacci V, Cambillau C, Ziebuhr J, Canard B 2006 Crystal structure and mechanistic determinants of SARS coronavirus nonstructural protein 15 define an endoribonuclease family. Proc Natl Acad Sci U S A 103:11892–11897. doi:10.1073/pnas.0601708103. 16882730 PMC2131687

[27] Bhardwaj K, Sun J, Holzenburg A, Guarino LA, Kao CC 2006 RNA recognition and cleavage by the SARS coronavirus endoribonuclease. J Mol Biol 361:243–256. doi:10.1016/j.jmb.2006.06.021. 16828802 PMC7118729

[28] Joseph JS, Saikatendu KS, Subramanian V, Neuman BW, Buchmeier MJ, Stevens RC, Kuhn P 2007 Crystal structure of a monomeric form of severe acute respiratory syndrome coronavirus endonuclease nsp15 suggests a role for hexamerization as an allosteric switch. J Virol 81:6700–6708. doi:10.1128/JVI.02817-06. 17409150 PMC1900129

[29] Kang H, Bhardwaj K, Li Y, Palaninathan S, Sacchettini J, Guarino L, Leibowitz JL, Kao CC 2007 Biochemical and genetic analyses of murine hepatitis virus Nsp15 endoribonuclease. J Virol 81:13587–13597. doi:10.1128/JVI.00547-07. 17898055 PMC2168834

[30] Posthuma CC, Nedialkova DD, Zevenhoven-Dobbe JC, Blokhuis JH, Gorbalenya AE, Snijder EJ 2006 Site-directed mutagenesis of the nidovirus replicative endoribonuclease NendoU exerts pleiotropic effects on the arterivirus life cycle. J Virol 80:1653–1661. doi:10.1128/JVI.80.4.1653-1661.2006. 16439522 PMC1367138

[31] Hagemeijer MC, Verheije MH, Ulasli M, Shaltiël IA, de Vries LA, Reggiori F, Rottier PJ, de Haan CA 2010 Dynamics of coronavirus replication-transcription complexes. J Virol 84:2134–2149. doi:10.1128/JVI.01716-09. 20007278 PMC2812403

[32] Freeman MC, Graham RL, Lu X, Peek CT, Denison MR 2014 Coronavirus replicase-reporter fusions provide quantitative analysis of replication and replication complex formation. J Virol 88:5319–5327. doi:10.1128/JVI.00021-14. 24623413 PMC4019139

[33] Graham RL, Sims AC, Baric RS, Denison MR 2006 The nsp2 proteins of mouse hepatitis virus and SARS coronavirus are dispensable for viral replication. Adv Exp Med Biol 581:67–72. doi:10.1007/978-0-387-33012-9_10. 17037506 PMC7123188

[34] Narayanan K, Makino S 2001 Cooperation of an RNA packaging signal and a viral envelope protein in coronavirus RNA packaging. J Virol 75:9059–9067. doi:10.1128/JVI.75.19.9059-9067.2001. 11533169 PMC114474

[35] Woo K, Joo M, Narayanan K, Kim KH, Makino S 1997 Murine coronavirus packaging signal confers packaging to nonviral RNA. J Virol 71:824–827. 8985424 10.1128/jvi.71.1.824-827.1997 PMC191125

[36] Kuo L, Masters PS 2013 Functional analysis of the murine coronavirus genomic RNA packaging signal. J Virol 87:5182–5192. doi:10.1128/JVI.00100-13. 23449786 PMC3624306

[37] Fosmire JA, Hwang K, Makino S 1992 Identification and characterization of a coronavirus packaging signal. J Virol 66:3522–3530. 1316465 10.1128/jvi.66.6.3522-3530.1992 PMC241133

[38] Chen SC, van den Born E, van den Worm SH, Pleij CW, Snijder EJ, Olsthoorn RC 2007 New structure model for the packaging signal in the genome of group IIa coronaviruses. J Virol 81:6771–6774. doi:10.1128/JVI.02231-06. 17428856 PMC1900089

[39] Yount B, Denison MR, Weiss SR, Baric RS 2002 Systematic assembly of a full-length infectious cDNA of mouse hepatitis virus strain A59. J Virol 76:11065–11078. doi:10.1128/JVI.76.21.11065-11078.2002. 12368349 PMC136593

[40] Klumperman J, Locker JK, Meijer A, Horzinek MC, Geuze HJ, Rottier PJ 1994 Coronavirus M proteins accumulate in the Golgi complex beyond the site of virion budding. J Virol 68:6523–6534. 8083990 10.1128/jvi.68.10.6523-6534.1994 PMC237073

[41] Lavi E, Wang Q, Weiss SR, Gonatas NK 1996 Syncytia formation induced by coronavirus infection is associated with fragmentation and rearrangement of the Golgi apparatus. Virology 221:325–334. doi:10.1006/viro.1996.0382. 8661443 PMC7131612

[42] Quinkert D, Bartenschlager R, Lohmann V 2005 Quantitative analysis of the hepatitis C virus replication complex. J Virol 79:13594–13605. doi:10.1128/JVI.79.21.13594-13605.2005. 16227280 PMC1262582

[43] Williams RK, Jiang GS, Holmes KV 1991 Receptor for mouse hepatitis virus is a member of the carcinoembryonic antigen family of glycoproteins. Proc Natl Acad Sci U S A 88:5533–5536. doi:10.1073/pnas.88.13.5533. 1648219 PMC51911

[44] Chen W, Madden VJ, Bagnell CR Jr, Baric RS 1997 Host-derived intracellular immunization against mouse hepatitis virus infection. Virology 228:318–332. doi:10.1006/viro.1996.8402. 9123839

[45] Zhou H, Perlman S 2007 Mouse hepatitis virus does not induce beta interferon synthesis and does not inhibit its induction by double-stranded RNA. J Virol 81:568–574. doi:10.1128/JVI.01512-06. 17079305 PMC1797428

[46] Smith EC, Case JB, Blanc H, Isakov O, Shomron N, Vignuzzi M, Denison MR 2015 Mutations in coronavirus nonstructural protein 10 decrease virus replication fidelity. J Virol 89:6418–6426. doi:10.1128/JVI.00110-15. 25855750 PMC4474304

[47] Eckerle LD, Becker MM, Halpin RA, Li K, Venter E, Lu X, Scherbakova S, Graham RL, Baric RS, Stockwell TB, Spiro DJ, Denison MR 2010 Infidelity of SARS-CoV Nsp14-exonuclease mutant virus replication is revealed by complete genome sequencing. PLoS Pathog 6:e1000896. doi:10.1371/journal.ppat.1000896. 20463816 PMC2865531

[48] Fleming JO, Shubin RA, Sussman MA, Casteel N, Stohlman SA 1989 Monoclonal antibodies to the matrix (E1) glycoprotein of mouse hepatitis virus protect mice from encephalitis. Virology 168:162–167. doi:10.1016/0042-6822(89)90415-7. 2535900 PMC7131138

[49] Gosert R, Kanjanahaluethai A, Egger D, Bienz K, Baker SC 2002 RNA replication of mouse hepatitis virus takes place at double-membrane vesicles. J Virol 76:3697–3708. doi:10.1128/JVI.76.8.3697-3708.2002. 11907209 PMC136101

[50] Schindelin J, Arganda-Carreras I, Frise E, Kaynig V, Longair M, Pietzsch T, Preibisch S, Rueden C, Saalfeld S, Schmid B, Tinevez JY, White DJ, Hartenstein V, Eliceiri K, Tomancak P, Cardona A 2012 Fiji: an open-source platform for biological-image analysis. Nat Methods 9:676–682. doi:10.1038/nmeth.2019. 22743772 PMC3855844

[51] Perlman S, Schelper R, Bolger E, Ries D 1987 Late onset, symptomatic, demyelinating encephalomyelitis in mice infected with MHV-JHM in the presence of maternal antibody. Microb Pathog 2:185–194. doi:10.1016/0882-4010(87)90020-9. 2853274 PMC7135528

