# Supplementary material for: In Situ Tagged nsp15 Reveals Interactions with Coronavirus Replication/Transcription Complex-Associated Proteins
Source: mBio. 2017 Jan 31;8(1):e02320-16. doi: 10.1128/mBio.02320-16 (PMC5285509; doi:10.1128/mBio.02320-16)
Supplement: TABLE S1 [file mbo001173167st1.docx]

| Gene | Primers |
| --- | --- |
| mHPRT Fwd | 5’-gcgtcgtgattagcgatgatg |
| Rev | 5’-ctcgagcaagtctttcagtcc |
| gRNA Fwd | 5’-agggagtttgaccttgttcag |
| Rev | 5’-ataatgcacctgtcatcctcg |
| sgRNA Fwd | 5’-tataagagtgattggcgtcc |
| sgRNA 7 Rev | 5’-ggcactccttgtccttgt |
| sgRNA 6 Rev | 5’-gtggccacattaaccacaag |
| sgRNA 5 Rev | 5’-agagggagacagcaacaaagt |
| sgRNA 4 Rev | 5’-cggcgttactggtgtacttg |

**Table S1**-List of primers
